# Immunometabolic Dysregulation at the Intersection of Obesity and COVID-19

**DOI:** 10.3389/fimmu.2021.732913

**Published:** 2021-10-19

**Authors:** Collins N. Khwatenge, Marquette Pate, Laura C. Miller, Yongming Sang

**Affiliations:** ^1^ Department of Agricultural and Environmental Sciences, College of Agriculture, Tennessee State University, Nashville, TN, United States; ^2^ Virus and Prion Research Unit, National Animal Disease Center, United States Department of Agriculture, Agricultural Research Service, Ames, IA, United States

**Keywords:** obesity, metabolic disorder, immunometabolism, COVID-19, immunopathy

## Abstract

Obesity prevails worldwide to an increasing effect. For example, up to 42% of American adults are considered obese. Obese individuals are prone to a variety of complications of metabolic disorders including diabetes mellitus, hypertension, cardiovascular disease, and chronic kidney disease. Recent meta-analyses of clinical studies in patient cohorts in the ongoing coronavirus-disease 2019 (COVID-19) pandemic indicate that the presence of obesity and relevant disorders is linked to a more severe prognosis of COVID-19. Given the significance of obesity in COVID-19 progression, we provide a review of host metabolic and immune responses in the immunometabolic dysregulation exaggerated by obesity and the viral infection that develops into a severe course of COVID-19. Moreover, sequela studies of individuals 6 months after having COVID-19 show a higher risk of metabolic comorbidities including obesity, diabetes, and kidney disease. These collectively implicate an inter-systemic dimension to understanding the association between obesity and COVID-19 and suggest an interdisciplinary intervention for relief of obesity-COVID-19 complications beyond the phase of acute infection.

## Association of obesity With the Severity of COVID-19

We are within an epidemic of obesity worldwide, and it has been getting worse. Applying a threshold that defines obesity by a body mass index (BMI) of 30 kg/m^2^ or greater, statistics across the globe indicate more than 1 in 10 adults (13.2%) were obese in 2019. This is equal to over 500 million obese people in total (1, 2). The United States is among the countries with the heaviest obesity burden with over 42% of the adult population meeting the criteria for obesity (2). Extensive clinical observations and epidemiological studies have shown that various metabolic disorders (including hyperlipidemia, hyperglycemia, hyperleptinemia, and insulin resistance) and multiple diseases (including diabetes mellitus, hypertension, cardiovascular diseases, cancers, and chronic kidney diseases) are often comorbid with obesogenesis as it becomes more severe with a BMI ≥ 40 (class III obesity) (2–5). Previous studies have demonstrated that viral infections such as those by some members of *adenoviridae*, *herpesviridae*, and *hepatitides* are frequently associated with obese individuals, who in turn show a higher disease risk to certain viral infections during influenza and dengue epidemics (6–9). A meta-analysis between obesity and influenza-related pneumonia during the 2009 influenza pandemic found that, compared with the normal BMI group, the risk of pneumonia in the obese (BMI ≥30) and morbidly obese (BMI ≥40) was raised 1.33 times (95% CI: 1.05–1.63) and 4.6 times (95% CI: 2.2–9.8), respectively (8). Furthermore, detailed in clinical studies of a hospitalized influenza virus-infected cohort, obesity was associated with increased risk for hospitalization, intensive care unit (ICU) admission, and invasive mechanical ventilation (IMV). Further still, morbid obesity (BMI >40) was linked to a twofold mortality risk from influenza virus infection (OR = 2.01, 95% CI: 1.29–3.14) (7–10). In addition, obesity increased the disease risk affected by influenza virus and prolonged the virus-shedding period for 42% longer than in non-obese controls (7–10). These meta-analytic studies of the risk relationship between obesity and common respiratory viral infections before the COVID-19 pandemic signify the comorbidity role of obesity in worsening viral pneumonia (8–10). The term “infectobesity” was proposed to define obesity of infectious etiology (5, 6). Whereas the infectious origin of obesity is still disputable, it becomes evident that obesity progressively undermines the inter-systemic homeostasis involving the metabolic, endocrine, neurological, and immune systems, which might underlie the vulnerability of the obese population to infectious risks to be fully understood (2–6).

Over a year after its outbreak, increasing clinical and epidemiological evidence shows that obesity is a major factor in increasing disease risk to severe acute respiratory syndrome coronavirus 2, SARS-CoV-2 (11–22). Obesity and its relevant metabolic disorders are among the top-ranked comorbidity associated with a severe course of COVID-19 resulting in higher rates of hospitalization, ICU admission, intubation (ITU) and IMV, and mortality. Several comprehensive reviews and meta-analyses studies (12–22) on the prevalence and prognosis of COVID-19 with obesity are referred, and this review will be focused on discussion of the immunometabolic dysregulation underlying this association. **Table 1** summarizes the major criteria in the association between obesity and COVID-19 from nearly 80 relevant studies, including approximately 0.5 million patients, qualified and adapted from several meta-analytic studies before March 2021 (12–32). We further compared based on the three world regions where almost all the relevant studies were conducted, i.e., Asia-Mideast, Europe, and America. Within the reported timeline of COVID-19 spreading around the world and the affected regional population, studies from China comprised the early 12 of 14 reports in the Asian-Mideast region including two other reports each from Singapore or Israel, followed by 14 from European countries and 35 studies from the U.S. In general, the patient cohort size of studies from the Asia-Mideast were small (<500 patients) except for the meta-analysis from Israel with a cohort size of 4,353. In contrast, there were four studies from Europe and 16 from the Americas with a cohort size greater than 1,000. In consensus of all the studies, obesity, especially severe obesity, has a strong positive association with at least one criterion defining severe COVID-19 progression, including the rate of hospitalization, ICU admission, ITU/IMV, and death. Nevertheless, the odds ratio (OR, a measure of association between an exposure and an outcome) used to estimate the association varies at a broad range of 1.1–9.2 due the heterogeneity of cohort sizes and patient demography among the studies. Cross-study data curation of eight studies with a cohort size >5,000 indicates a conservative OR range at 1.8–3.2 for increasing COVID-19 severity and mortality by obesity and associated comorbidities (**Table 1** and **Supplement Excel Sheet**) (12–32).

**Table 1:** Summaries with obesity as a risk factor for the prevalence, prognosis and/or sequela of COVID-19 (12–32).

World region/Country	Asia-Mideast	Europe	Americas	Summary
**• Number of studies**	14 (12 China, 1 Singapore, and 1 Israel)	14 (3 France, 7 Italy, 2 UK, and 1 Germany/European	35 (29 USA, 5 Mexico, and 1 Brazil)	63
**• Study design**	Cross-sectional/Cohort studies of hospitalized	Cohort /Cross-sectional studies of hospitalized or dead	Cross-sectional/Cohort studies of hospitalized or diagnosed	
**• Number of participants (Range; >1000 studies)**	6,754 (58-4,353; 1)	29,740 (50-20,133; 3)	436,306 (103-212,802; 16)	472,800 (20)
**• Age group (Ranges of the Median/mean)**	38-61 yr	60-72.9 yr	43-68 yr	
**• Males (%)**	47.8-74.2%	52-73%	37.5-64.8%	
**• Prevalence of COVID-19 with obesity**	10.7-68.2%	10.5-65.2%	14-66.5%	
**• Prognosis of COVID-19 with obesity (OR, odd ratio)**	BMI/Obesity positively associated with: Severity: Pneumonia (1.3), overall severe illness (1.1-9.2)	Severity: Hospitalization (2.2), ARDS (2.0-2.3), ICU (2.0-4.9), IMV (2.1-7.4); overall severe illness (1.4) Mortality (1.3-3.0);	Severity: Hospitalization (1.3-4.4), ARDS (2.4), ICU (1.3-2.2), ITU (1.4), IMV (2.1-4.5) Mortality (1.2-2.3)	Ave OR: 3.2* Ave OR: 1.8*
**• Complications of Obesity/COVID-19 (%, Odds ratio to COVID-19)**	Hypertension (14-15%, 4.6); diabetes (6-20%, 3.2); CVD (9%, 2.8)	Hypertension (18.4-77.2%, 4.6); diabetes (23-58%, 3.2); CVD (28-55%)	Hyperlipidemia (46%), hypertension (15-80%); diabetes (16-53%, 1.3-2.5); CVD (19-35%, 1.3)	Ave OR: 2.9*

*Ave OR: The average of listed Odds Ratio (OR) without inclusion of the lowest/highest OR values of studies with >5,000 participants. ARDS, acute respiratory distress syndrome; CVD, cardiovascular diseases; ICU, intensive care unit; IMV, invasive mechanical ventilation; ITU, intubation.

**Figure 1:**
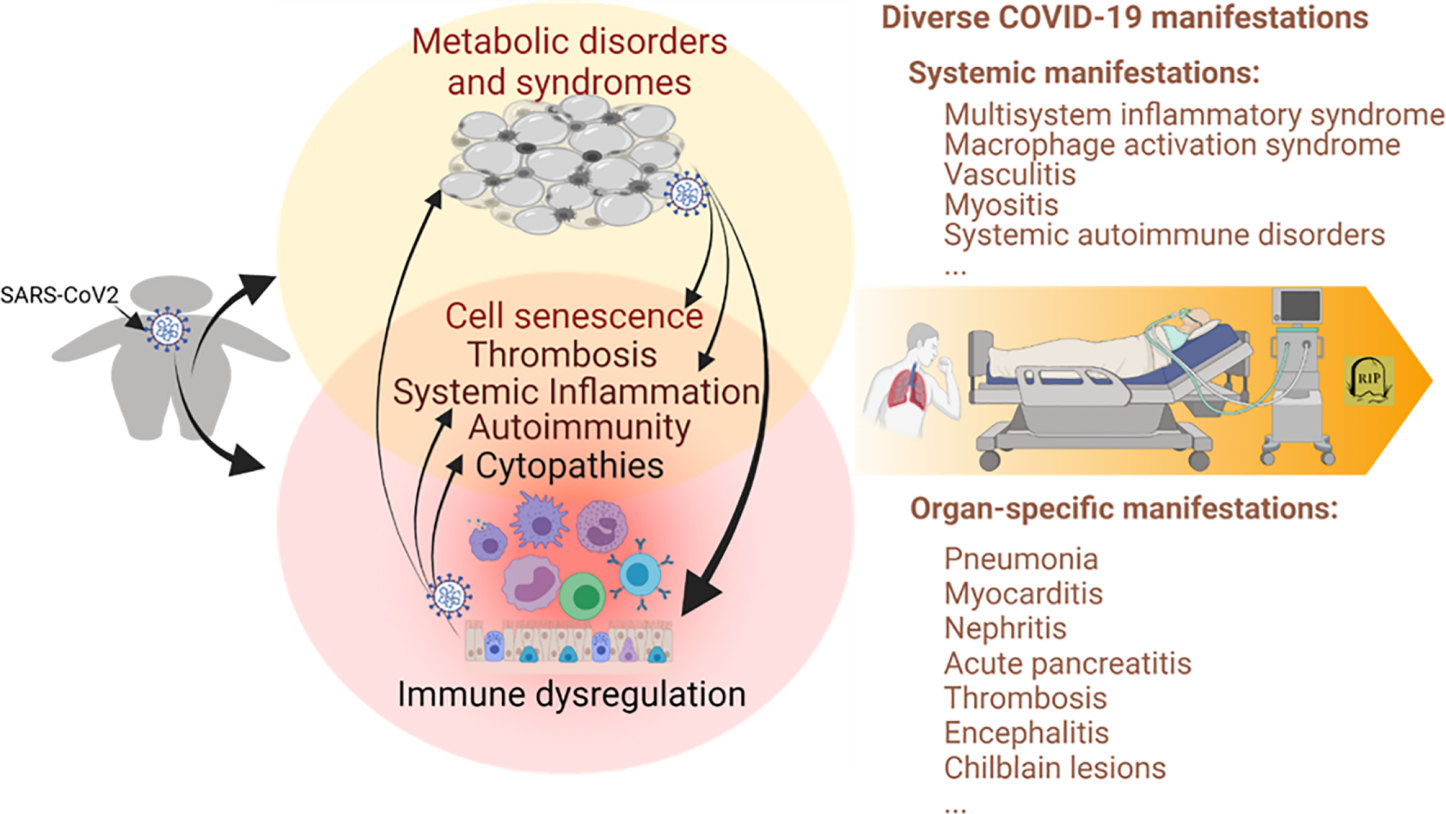
Immunometabolic proxies underlying the association between obesity and the severe course of COVID-19. Studies have associated the propensity of severe COVID-19 manifestation in patients with obesity and relevant metabolic comorbidities. The emerging theme indicates the immuno-pathological exaggeration of predisposed immuno-metabolic disorder (indicated in the circles, especially the overlapped region on the left) in the obese COVID-19 patients. This predisposed immunometabolic disorder magnifies, locally and systemically, the virus pathogenicity and relevant immunopathies leading to more severe COVID-19 manifestations. COVID-19, coronavirus disease 2019; SARS-CoV-2, severe acute respiratory syndrome coronavirus 2. Created with BioRender.com.

About half of the studies compared virus prevalence in obese with non-obese individuals, indicating a higher ratio at 10.5%–68.2% of positive diagnosed in the obese population considering that the average country-wide infection ratio of SARS-CoV-2 is 2%–10% (**Table 1** and **Supplement Excel Sheet**) (12–32). The higher prevalence of COVID-19 reflects a generally more susceptible status and slower recovery process of the virus infection in the obese population, who, in other words, have a compromised defensive response to restrict and clear the virus. Almost all the studies observed a strong relationship of severe COVID-19 with complications of obesity (BMI≥40), including primarily hyperlipidemia, hypertension, diabetes and cardiovascular diseases (CVD) (**Figure 1**) (4, 5, 33). Other factors, including gender and age, have been shown to affect COVID-19 progression and were included in the studies. For instance, obesity may increase COVID-19 risk for young patients, who are generally more resistant to developing severe COVID-19 (11–22, 34–37).

In summary, this review unifies the most relevant studies and meta-analytic data to reinforce that obesity, especially the severe obesity with morbid complications, represents a consistent risk factor underlying the prevalence and particularly severe manifestations of COVID-19 (**Table 1** and **Figure 1**) (12–32). It is of note that obesity defined by the BMI threshold does not have to correlate to a morbid condition in every obese individual. A study examining 247 COVID-19 patients showed that higher levels of cardiorespiratory fitness (CRF) are associated with lower hospitalization rates, suggesting that fitness is more critical than adiposity in predicting the risk for COVID-19 hospitalization and potential for severe COVID-19 (38). Of note, the immunometabolic mechanism underlying the association between obesity and COVID-19 is just emerging as reviewed below and awaits further investigations in order to develop therapeutic intervention at the immunometabolic interface (4, 18, 34, 39, 40).

## Immunometabolic Proxies Underlying the Complication of Obesity and Severe COVID-19

During the acute phase, about 4 weeks after respiratory infection by SARS-CoV-2, COVID-19 manifests a wide range of symptoms in people influenced by different demographic factors including age, sex, ethnicity, and, particularly, pre-existing medical conditions including obesity and associated metabolic syndromes (33, 41). Studies in virology and epidemiology indicate that SARS-CoV-2 evolves in a balance between virulence and contagiousness to spread efficiently in people, while posing morbid and mortal threats in vulnerable subgroups (42, 43). The prognosis of COVID-19 patients has a broad spectrum: with the vast majority (50%–80% per different research scenarios, CDC) only having mild symptoms like a common cold or asymptomatic, and the other 20%–50% developing into severe local and systemic syndromes needing immediate hospitalization and critical care (41, 44–46). The death rate of COVID-19 varies at 1.7%–13.0% due to heterogeneous demographics and intervention in different countries (41, 44). Examining approximately 3,000 reported cases worldwide, Ramos-Casals et al. profiled more than 70 different systemic and organ-specific morbidities during the severe course of COVID-19 (**Figure 1**, right part) (33, 41). In addition to the pathogenic impact triggered by local viral infections such as airway inflammation and pneumonia, major pathologies underlying severe COVID-19 come from the excessive immune response (immunopathies) (33). These immunopathies are very diverse but reflect common signs of autoimmune or inflammatory diseases as frequently observed in, but not limited to, morbid obesity and associated metabolic syndromes as reviewed elsewhere and briefed here (47–49). During COVID-19 progression, the virus–host interaction in humans has several characteristics, which highlight the stages of pathogenesis and potential cross-link underlying the association with obesity. First, the viral spike protein (S) interacts with its primary cell receptor, angiotensin-converting enzyme 2 (ACE2) expressed in multiple systems including adipose tissues (50–63). Functionally speaking, the S-binding of the ACE2 catalytic region suppresses ACE2’s role in angiotensin release and disrupts the body’s renin–angiotensin–aldosterone system (RAAS) leading to hypertension, vasoconstriction, inflammation, fibrosis, thrombosis, and pulmonary damage (64–66); these complications are also commonly observed in patients of morbid obesity. The viral effect in suppression of the host antiviral immunity (48, 67, 68), and induction of cell death in infected epithelia/endothelia and bound platelets by the virus, causes excessive pro-inflammation and amplifies into a cytokine releasing syndrome (CRS) (33, 69). The scale and intensity of the virus–host interaction are heterogeneous and lead to diverse manifestations of COVID-19, ranging from benign/self-limiting signs to life-threatening syndromes locally in the lung or inter-systemically in several organs (33, 41), which can be associated with and complicated by an obese precondition.

Obesity and associated metabolic syndromes are chronic comorbidities initiated by progressive accumulation of pathological adipose tissues (3–5, 70). Metabolic disorders increase local and plasma active mediators or metabolites, including insulin, glucose, lipids, leptin, and adiponectin, reflecting early diagnostic abnormalities (3–5, 70). Accompanying the morbid progression, metabolic disorders are exaggerated by both local and systemic distortion, especially dysregulated immunity involving chronic inflammation, increased thrombotic activity, and autoimmune inclinations (3, 70–72). Nevertheless, on a chronic and sub-intensive scale, this series of consequential immune diversion in obesity may build up an immunopathological propensity to potentiate the viral infection into a severe course of COVID-19 (10–13). As in the local and systemic manifestations of severe COVID-19 cases, morbid obesity and associated metabolic syndromes show comparable (but low-grade and chronic) immunopathies that involve destructive activity from systemic inflammation to increasing thrombotic and autoimmune disorders (3–5, 73–76). In turn, morbid obesity can be complicated clinically into hypertension, CVD, respiratory, renal, and hepatic diseases in severe COVID-19 cases as a function of patient-dependent acute phase (12–18, 33, 41). **Figure 1** illustrates the immunometabolic proxies underlying the association between obesity and severe COVID-19, recapping the theme of the immuno-pathological predisposition of obesity toward the severe progression of COVID-19 (12–18, 33, 41). Notably, recent studies also highlight a cell senescence process involving increased cell defects of both visceral epithelial and mesenchymal cells affected by pathological adipose tissues. This obesity-related cell senescence increases the virus susceptibility, amplifies proinflammatory signaling, suppresses IFN responses, and incites the virus-mediated cell death during COVID-19 progression in obese patients (77–83). Collectively, obesity and SARS-CoV-2 infection, differently initiated from either metabolic or immune dysregulation, show aggregation in common immunopathies contributing to the severe course of COVID-19 and demonstrate epidemiological association where the obesity epidemic unfortunately meets the COVID-19 pandemic in our era (**Figure 1**) (3–13).

## Obesity-Originated Immunometabolic Diversion and COVID-19

This section examines immunometabolic dysregulation and derived immunopathies from the obesogenic angle of COVID-19 in obese patients. Note that classical studies (except those specifically mentioned) of immunometabolic dysregulation in obesity were not conducted in cohorts with COVID-19 complications and thus may be referred to as rationally justified instead of direct evidence (5, 70–83).

**Figure 2:**
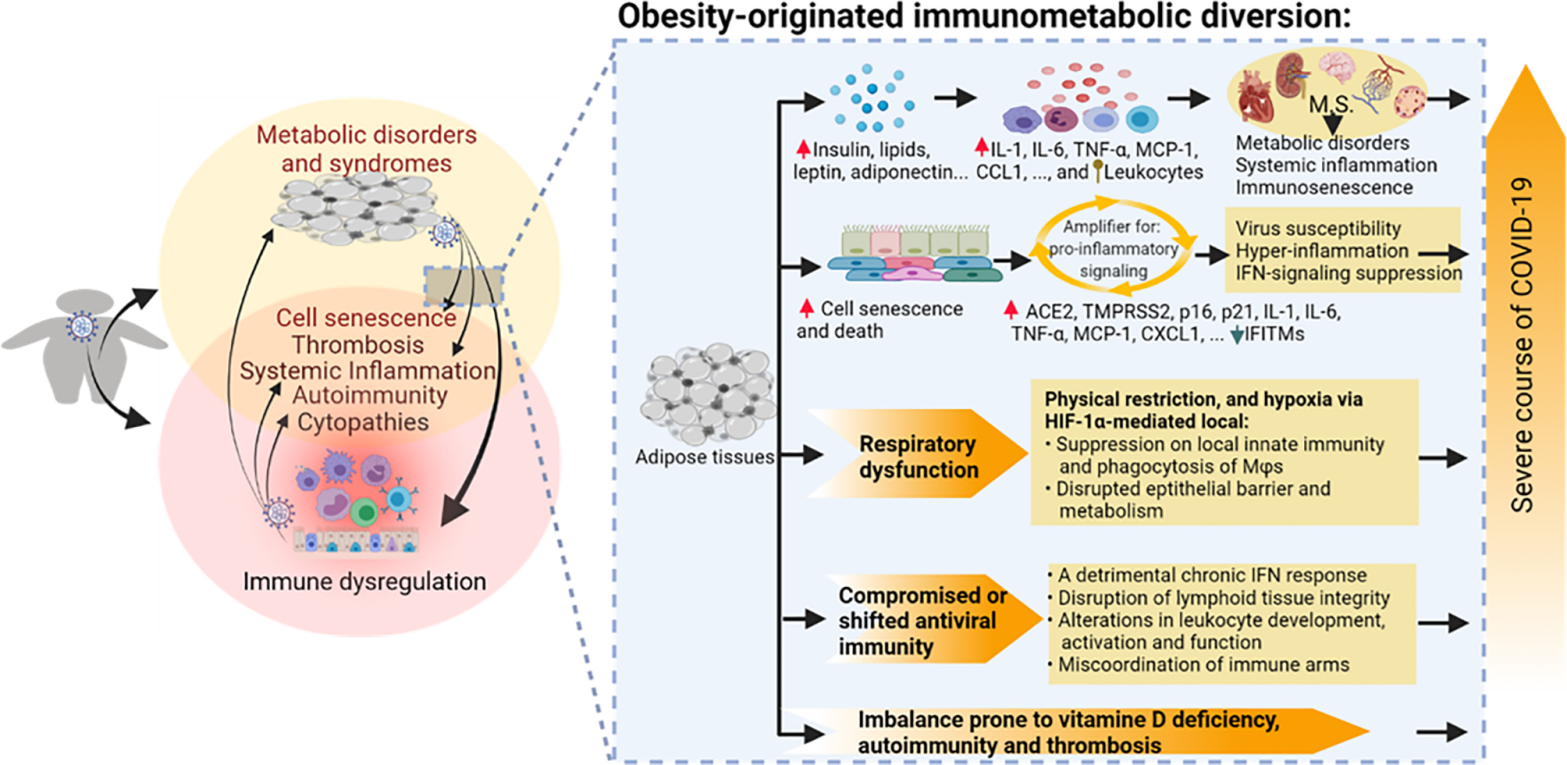
Obesity-originated immunometabolic diversion underlying the association between obesity and the severe course of COVID-19 manifestations. Studies about obesity and associated metabolic disorders show the following: (1) increases of local and plasma mediators including leptin, insulin, glucose, lipids, and particularly various proinflammatory cytokines/leukocytes underlying relevant metabolic stress and inflammatory status accompanying excessive deposit of adipose tissues and development of metabolic disorders; (2) high incidences of cell senescence and death of adipocytes and associated epithelial and mesenchymal cells, which amplify proinflammatory signaling, dysregulate IFN-mediated antiviral response, and induce tissue expression of molecules facilitating SARS-CoV-2 susceptibility; (3) compromised or shifted antiviral immunity in both innate and adaptive arms; (4) respiratory obstruction resulting in hypoxia-mediated immune impairment; and (5) imbalance prone to autoimmune and thrombotic response. Arrowheads indicate increase (upward red) or decrease (downward green) of following molecular markers. Upward ball-head symbol ascribing abnormal chemotaxis and activation on leukocytes by obesity. ACE2, angiotensin converting enzyme 2; CCL, CC type chemokine ligand; CXCL, CXC type chemokine ligand; IFN, interferon; IFITM, interferon-induced trans-membrane proteins; IL, interleukin; MCP-1, monocyte chemoattractant protein-1; p16 and p21, cellular senescence marker protein 16 and 21; TMPRSS2, transmembrane Serine Protease 2; TNF, tumor necrosis factor.

As shown in **Figure 2**, adipose tissues serve as an endocrine organ secreting adipokines, including leptin, adiponectin, visfatin, and resistin (72, 83–87). These adipokines act on different tissues and cells to regulate energy homeostasis and metabolism and mediate inflammation, immune response, and tissue damage. With the exception of adiponectin, the other three adipokines, i.e., leptin, visfatin, and resistin, are proinflammatory and their levels increase during obesity (85–89). Hyperleptinemia, ascribing an elevated leptin level in circulation, is among the diagnostic criteria of hyperlipidemia, hyperglycemia, and hyperinsulinemia (insulin resistance), indicating a metabolic disorder of obesity and associated syndromes (3–5, 10, 72). Studies have shown an important role of obesogenic leptin (similarly for less studied visfatin and resistin) in inflammatory and immunometabolic regulation. Hyperleptinemia in obesity or associated comorbidities is associated with, locally or systemically, increases of proinflammatory cytokines including interleukin (IL)-1β, IL-6, IL-8, IL-12, and tumor necrosis factor (TNF)-α (87–90). Leptin mediates chemotactic infiltration and activation of innate immune cells including monocytes/macrophages, neutrophils, and natural killer cells (NK) to increase cytotoxicity (72, 89–92). Visceral fat tissue takes a greater part in obesity and produces highly proinflammatory cytokines, including IL-6, TNF-α, and IL-8, compared to its subcutaneous counterpart (88–91). In adaptive immunity, leptin induces the proliferation of naïve T cells and B cells while reducing the generation of immunosuppressive regulatory T cells (Treg). In T-cell activation, leptin switches T helper (Th) cells towards pro-inflammatory Th1/Th17 rather than the anti-inflammatory Th2 phenotype. Finally, leptin activates B cells to secrete cytokines and modulates B-cell development (89–92). The improper stimulation of immune responses in obesity without timely resolution perpetuates obesity-associated inflammation and cell/tissue damage. Adipocyte cell death at early stages during adipose tissue expansion triggers the inflammation and recruitment of immune cells to adipose tissue; however, studies demonstrated cell senescence and death accounting for amplification of proinflammation and antiviral suppression in visceral organs associated with aging and obesity in severe COVID-19 progression (77–84).

Obesity triggers cellular senescence extending from adipocyte decay to affected visceral endothelial, epithelial, and mesenchymal cells in various metabolic syndromes (77–84). Obesity- and aging-induced cell senescence has been associated with inflammation underlying multiple chronic diseases (84, 93). Using engineered mouse models, Camell et al. demonstrated that these senescent cells became hyper-inflammatory in response to SARS-CoV-2 infection and in the presence of the viral spike protein (84). This led to consequential increased expression of viral entry proteins including ACE2 and transmembrane serine protease 2 (TMPRSS2) and reduced the antiviral IFN-stimulated gene expression in surrounding endothelial cells. Depleting senescent cells using senolytic drugs significantly reduced the inflammatory markers, increased antiviral antibodies, and increased the survival rate in vulnerable mice following the coronavirus infection (84). This study thus correlates cell senescence, a common pathological mechanism underlying aging, obesity, and other preexisting comorbidities that may be amplified during SARS-CoV-2 infection to increase virus spreading, CRS, and other immunopathology in severe COVID-19 cases (**Figures 1**, **2**) (77–93).

The physiological milieu of obesity and metabolic syndromes may undermine the human immune system directly through altering lymphoid tissue architecture and integrity, changing leukocyte activation and inflammatory phenotypes, and impairing effector T-cell and B-cell specificity in resolving infection from pathogens, including respiratory viruses (5, 94–98). Compared with healthy controls, COVID-19 patients have a pro-inflammatory activation of lung macrophages and blood monocytes, a lower blood leukocyte count particularly that of T cells, and improper activation of NK and T cells (99). Prominent in lymphopenia, this immune dysfunction has a greater association in severe COVID-9 patients with pneumonia than those who just have mild or asymptomatic signs (99–108). Although few studies have demonstrated if obesity-related immune dysregulation directly contributes to immune disruption by SARS-CoV-2, the high association of obesity with severe COVID-19 cases indicates this potential. A recent study about memory B cell (MBC) response indicated that unlike in young patients who had effective B cells specific to SARS-CoV-2 spike protein, which are protective, older patients had more MBCs targeting the other viral proteins, which are not protective (109). This should be similar in obese COVID-19 patients, given that delayed bystander CD8 T cells and less specific B cell response to SARS-CoV-2 were observed (99, 108, 109). In addition to cell immunity, obesity also progressively alters the antiviral IFN response of the innate immune arm, which shows persistent IFN production with a detrimental role, rather than antiviral protection during the acute phase of a viral infection (5–7).

Biomechanically, the excess of intrapulmonary and pericardial fat in morbid obesity impairs gas exchange and respiratory mechanics through lowering lung volume, weakening respiratory muscle strength, and increasing airway resistance (110–115). This worsens due to the inflammatory swelling and the hypersensitive reaction induced by proinflammatory adipokines and cytokines during infection (112, 114). An increase in plasma leptin levels and inflammatory mediators were found in severe COVID-19 cases with pulmonary symptoms (114, 115). The intrapulmonary adipocytes may serve as direct target cells for SARS-CoV-2 infection and amplify the proinflammatory effect to aggravate inflammatory infiltrates and trigger massive interstitial edema (57, 58, 111). In addition, a hypoxia environment will be induced in the inflamed and infected lung, which, in turn, activates signaling pathways mediated by hypoxia-induced factor 1α (HIF-1α). Hypoxia and the activation of HIF-1α-mediated signaling may alter an array of physiological and immune functions at the airway epithelial interface and have recently been proposed to serve as therapeutic targets for COVID-19 intervention (116, 117). In this context, several studies showed that patients subjected to IMV had a higher visceral fat index (10, 118). Other host responses connecting obesity and COVID-19 severity include disorders prone to vitamin D deficiency, thrombosis, and autoimmunity against self-antigens, which are consequences of metabolic and immune diversion posed by obesity or coronaviral infection (119–121). Vitamin D and 25-hydroxycholesterol (25HC) represent two metabolites in cellular cholesterol metabolism prominent for their regulatory role in both host metabolism and immune reactions (119–121). As 25HC exerts a broad antiviral activity, vitamin D has been recommended as a nutritional supplement to the prevention of type 2 diabetes (T2D) and COVID-19 progression (119, 122). Endothelial dysfunction and thrombosis are complicated in morbid obesity and particularly in cardiometabolic syndrome (73–76). SARS-CoV-2 causes endothelial infection and manifests serial symptoms associated with thrombosis as shown in **Figure 1** and extensively reviewed (123–125). Autoimmune reactions indicate a functional indeterminate in the antibodies**’** specificity against self- and non-self-antigens, which again have been associated with various metabolic syndromes and demonstrated to be a common diagnostic criterion for about 15% of severe COVID-19 cases (48, 126).

In summary, obesity and associated metabolic disorders may exacerbate the susceptibility and severity of COVID-19 through the following aspects: (1) increases of local and plasma mediators, including insulin, lipids, leptin, adiponectin, and particularly various proinflammatory cytokines and leukocyte infiltrates (10, 72, 91, 92); (2) high incidences of cell senescence of adipocytes and surrounding visceral cells, which amplify proinflammatory signaling, dysregulate IFN-mediated antiviral response, and increase proteins to promote SARS-CoV-2 susceptibility (80–84); (3) compromised or shifted antiviral immunity in both the innate and adaptive arms (10, 72, 91, 92); (4) respiratory obstruction resulting in hypoxia-mediated immune diversion (110–117); and (5) imbalance prone to autoimmune and thrombotic response (**Figure 2**) (123–126).

## The Virus–Immunity Interaction Intersecting to Obesity

As briefed above, SARS-CoV-2 demonstrates a broad cell/tissue tropism in humans. The epithelial cells lining the eyes and various visceral organs (including the lung, heart, intestines, kidney, liver, pancreas, and testicle) have been shown be susceptible to the virus infection (50–63), as are endothelial cells of the blood vessels, neuron cells in the brain, and possibly adipocytes in visceral fats (50–63, 127, 128). Human visceral epithelial cells, like certain airway and gut epithelial cells, are naturally susceptible due to constitutive expression of ACE2 and TMPRSS2 that facilitate SARS-CoV-2 entry into cells (50–63). In addition to widely expressed ACE2 and TMPRSS2 as primary susceptibility factors, neuropilin1 (NRP1) was recently identified as an important cofactor to promote the virus entry in cells with low-level ACE2 expression (60). This may extend susceptible cell types and contribute to multi-organ symptoms in severe COVID-19 complications (33). The putative susceptibility of both adipocytes and endothelial cells indicates a nasty potential for SARS-CoV-2 spread through these connective tissue cells to trigger systemic symptoms of COVID-19 (**Figure 1**) (57, 58, 127, 128). Recent studies also indicated that the human ACE2 gene can be induced through epigenetic regulation by chronic IFN and inflammatory signaling (48, 129), implying that these cells can become more susceptible during severe COVID-19 progression in obese patients who have low-grade inflammation and detrimentally chronic IFN responses (48, 129–132). Therefore, inflamed adipocytes and endothelial cells from aging and obese individuals seem more permissive and vulnerable to virus infection, in turn boosting relevant immunopathies to exacerbate a severe course of COVID-19 (10–18).

**Figure 3:**
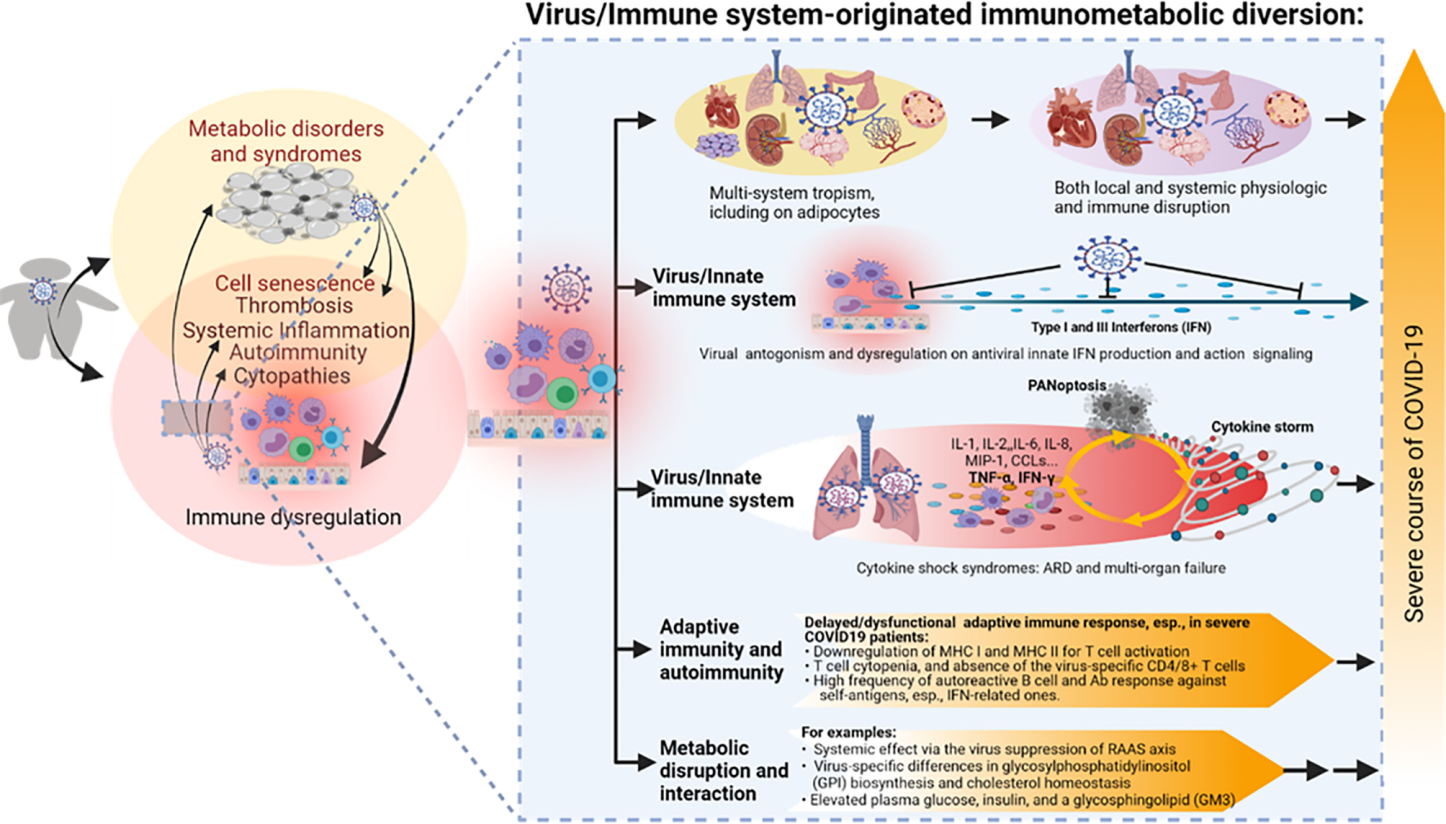
The virus–immune system interaction and relevant immunometabolic dysregulation underlying the association between obesity and the severe course of COVID-19. SARS-CoV-2 is notorious for its broad tissue tropism, including susceptibility that has been demonstrated in the airway, lung, intestine, kidney tubule, pancreatic duct, blood vessel, neuron, and adipocyte cells, implicating metabolic and immune disruption caused by direct viral infections in multiple systems. Studies indicate an extensive virus-immune system interaction at both innate and adaptive immune levels, with emphasis on the viral antagonism against the antiviral IFN system, hyper-inflammation incited by massive cell death upon immunopathies induced by the virus–host interaction, as well as various disruptions in adaptive immunity per T-cell- and B-cell-mediated responses. Some metabolic disruption of lipid biosynthesis has also been correlated to COVID-19 progression, but its long-term effect on the development of metabolic disorders in convalescent patients warrants further studies. PANoptosis, massive inflammatory cell death including Pytoptosis, Apoptosis, and Necroptosis. Other abbreviations are as listed in **Figure 2** legend.

In addition to the immune senescence and immunometabolic disruption as examined from the obesity perspective, studies have shown that the human innate immune system and its interaction with SARS-CoV-2 are early contributors to the heterogeneous disease courses of COVID-19 (33, 37, 40, 48, 67, 68). Plausibly, the difference between obese and non-obese populations about these innate immune components and their responses to the virus infection attributes much of immunometabolic dysregulation underlying the association of obesity and severe COVID-19 (10–18). Most studies on SARS-CoV-2 and its innate immune responses were conducted in patient cohorts or animal models without special consideration of the obesity factor (33, 37, 40, 48, 67, 68). First, virus tropism and its ability to subsist with the host**’**s innate immune response determine if the infection will be productive and develop into symptomatic disease (67, 68). As an RNA virus, SARS-CoV-2 may be recognized by cellular pathogen-pattern recognition receptors (PRRs) that sense foreign RNA including endosomal TLR3 and TLR7, as well as by cytoplasmic RIG-I-like receptors (RLR), including RIG-I and MDA5 (67, 68). The effective antiviral signaling mediated by these viral RNA receptors will lead to the production of inflammatory mediators and antiviral IFNs. IFNs will act to induce an antiviral state for local restriction of the infecting virus and inflammatory mediators will work to orchestrate acute inflammatory responses to bring in leukocytes and prepare for the activation of a specific immune response as needed to clear viruses evading the innate immune surveillance (33, 37, 40, 48, 67, 68, 133). Immune responses can be a double-edged sword and detrimental to the host unless properly regulated (67, 68); in IFN and inflammatory responses, both overactivation and underactivation can be deleterious to the host (48, 134). Early studies detected the impaired type I IFN responses in both SARS-CoV-2-infected human bronchial cells and circulating mononuclear blood cells early in infection (135, 136). Using plasma samples from hospitalized COVID-19 patients, lower IFN content was detected together with persistent viral genes and increased inflammatory cytokines including TNF-α and IL-6 (136). Other studies by Lee et al. (2020) and Lucas et al. (2020) detected that those patients with severe COVID-19 pneumonia endured a consistent pro-inflammatory response, but a peak of IFN response in their blood samples (137, 138). These results seem contradictory; however, they may reflect a viral suppression of IFN response at the acute phase of infection, and an immunopathological IFN induction compounded by inflammation and cell death in severe COVID-19 cases (135–138). The suppression of IFN response was further validated by the identification of at least 10 SARS-CoV-2 proteins targeting multiple steps across the IFN production and action signaling pathways (33, 48, 67, 139). In addition, recent genome-wide association studies (GWAS) associated severe COVID-19 incidences with several critical genetic regions, spanning multiple genes centered in both chemokine and IFN signaling (140, 141). All of these studies, from both the virus and host perspectives, highlight the determinant role of IFN and chemokine signaling in host susceptibility to SARS-CoV-2 infection and the progression of severe COVID-19 (135–141). Similarly, key genes in IFN and chemokine signaling have also been associated with obesity and metabolic syndromes including T2D, systemic lupus erythematosus (SLE), and CVD (10, 72, 91, 92). This demonstrates a cross-link of immune dysregulation in obese COVID-19 patients but awaits mechanistic studies (**Figure 3**).

Excessive production of pro-inflammatory cytokines and acute tissue damage in the lung mark the severe progression of COVID-19, albeit with systemic manifestations in other organs as well (33, 67). Multiple inflammatory cytokines and chemokines have been analyzed to highlight key players in determining SARS-CoV-2 severity. Profiling COVID-19 patient blood samples, Karki et al. detected 10 cytokines (including IFN-γ, IL-1α/β, IL-2, IL-6, IL-15, IL-18, IL-33, and TNF-α/β) that were upregulated in patients with moderate or severe COVID-19 compared with the samples from healthy individuals (69). Further tests in both cultured human cells and human ACE2-transgenic mice have highlighted the combination of TNF-α and IFN-γ induced inflammatory cell death coined as PANoptosis, which includes three types of cell death processes as pyroptosis, apoptosis, and necroptosis. The signaling cascade mediated by TNF-α and IFN-γ through the JAK/STAT1/IRF1 axis activates nitric oxide production amplifying the cytokine storm and cell death cycles, which mirror tissue damage and CRS observed in severe COVID-19 cases. Inhibiting PANoptosis using cell death inhibitors or treating with neutralizing antibodies against TNF-α and IFN-γ protected mice from mortality during SARS-CoV-2 infection as well as immunopathies including sepsis, hemophagocytic lymphohistiocytosis, and cytokine shock (69). This proof-of-the-concept study thus suggests a therapeutic potential to relieve the cytokine-mediated inflammatory cell death through intervening in the early TNF-α and IFN-γ combinative signaling through the JAK/STAT1/IRF1 axis. Notably, this newly discovered inflammatory cell death signaling associated with severe COVID-19 resembles a non-canonical IFN signaling that similarly compounds TNF-α and IFN-α/β to activate the IRF1/NF-κB/PU.1 axis in increased immunopathies, as observed in inflammatory and autoimmune disorders underlying morbid obesity and metabolic syndromes (69, 129, 132). This suggests that the signaling of TNF-α plus IFN-α/β combination is involved in a chronic inflammatory progression such as in obesity, but TNF-α plus IFN-γ combination works in a more acute/severe infectious situation such as in severe COVID-19 (69, 129, 132). It is therefore intriguing to investigate the relationship and cross-talk between these two proinflammatory signaling pathways both involving TNF-α potentiation but differing in IFN types of innate or adaptive immunity, respectively (69, 129–132).

In addition to the stratified dysregulation at the innate immune level by both the obesity and virus–host interaction, profound diversion of adaptive immune responses by SARS-CoV-2 infections has also been demonstrated in COVID-19 patients, particularly the severe cases (67, 68, 128, 133). First, SARS-CoV-2 is capable of suppressing the antigen presentation process to impede the activation of an adaptive immune response. A recent study showed that the ORF8 protein of SARS-CoV-2 can directly bind to the major histocompatibility complex I (MHC I) and redirect to a degradation pathway intracellularly before MHC I presenting antigens on the infected cell surfaces can activate CD8+ cytotoxic T cells (142). This study also showed that ORF8 of SARS-CoV-2 downregulates MHC I in cells; therefore, ORF8 protein has been proposed as a rapidly evolving accessory protein of coronaviruses that interferes with immune responses (142). In addition, the viral Nsp10 protein impedes antigen intracellular processing and loading to MHC II expressed by professional antigen presentation cells (including B cells, macrophages, and dendritic cells), which, in turn, suppress the activation of CD4+ T helper cells (143–145). Examination of clinical samples showed that an increase of dysfunctional classical monocytes with high expression of S100 protein and low MHC II expression was associated with severe COVID-19, reminiscent of the immunoparalysis seen in sepsis and other metabolic syndromes (133). A second diversion of the adaptive immune response by SARS-CoV-2 is T-cell atrophy or absence of the virus-specific CD4+ T cells in severe COVID-19 patients. Studies showed that induction of SARS-CoV-2-specific CD4+ T cells was highly associated with a good prognosis in COVID-19 severity. In contrast, the delay or absence of SARS-CoV-2-specific CD4+ T cells predicted a severe or fatal COVID-19 (107, 133, 146, 147). For B-cell-mediated humoral immunity, a striking observation highlighted by recent studies is that autoreactive antibodies, especially those specific against IFNs, were detected at a higher ratio (~15%) in severe COVID-19 patients than in the healthy controls (48, 126, 148). It also indicates that the gender factor contributes to the high autoimmune incidence, as men infected by the virus were more prone to develop into severe COVID-19 than women (48, 126, 148). Although autoimmunity is also commonly diagnosed in cases of morbid obesity and relevant complications, the autoimmune intensity and auto-antigen overlap between obesity and COVID-19 need to be studied using a comparative cohort including at least obese control and obese COVID-19 groups (**Figure 3**). Finally, the RAAS, which regulates multiple aspects of human physiological homeostasis, is also affected by SARS-CoV-2 infection (64–66, 149). In addition to its role in circulation and immune regulation, RAAS is intrinsic to tissues that modulate food intake, metabolic rate, adipogenesis, and insulin level/sensitivity (64–66, 149). Dysregulation of RAAS increases the production of inflammatory cytokines and reactive oxygen species (ROS) and exacerbates the insulin resistance that mediates glucose/lipid metabolism (64–66, 149). Studies using genetic analysis and pharmaceutic inhibitors have associated the dysregulated RAAS with metabolic syndromes such as type 2 diabetes (64–66, 149). Given SARS-CoV-2**’**s direct binding and suppression effect on ACE2, the metabolic aberration in the samples of COVID-19 patients is to be expected. For example, Song et al. performed quantitative lipidomic and metabolomic analyses using plasma samples from COVID-19 patients (150). They showed that the plasma metabolite panel effectively distinguished COVID-19 patients from healthy controls. In particular, the level of a type of sphingolipids (i.e., monosialodihexosyl gangliosides, GM3s) in the plasma was negatively correlated with CD4+ T-cell count in COVID-19 patients, and accordingly, the plasma from severe patients contained GM3-enriched exosomes (150). Given the important role of sphingolipids in the regulation of T-cell proliferation and activation, this study correlates lipid metabolism to T-cell-mediated immunity with the severe course of COVID-19 (151, 152). Yu et al. demonstrated that insulin therapy itself might increase mortality in patients with COVID-19 and diabetes, remarking the shared biomarkers of increased plasma insulin and glucose in both obese and severe COVID-19 patients (153). Hoffmann et al. conducted protein interactome screening in SARS-CoV-2-infected human cells and found virus-specific differences in glycosylphosphatidylinositol (GPI) anchor biosynthesis and multiple pan-coronavirus factors involved in cholesterol homeostasis (154). Both GPI and cholesterol biosynthesis have been implicated in the obesogenic process and antiviral immune regulation but have not been studied for regulation in the severe course of COVID-19 in obese patients (154).

Collectively, beyond the extensive association analyses between obesity and COVID-19, there are limited studies probing the immunometabolic dysregulation in obese COVID-19 patients (151–154). Several potential cross-links include the following: (1) SARS-CoV-2**’**s broad tissue/cell tropism with the emphasis of susceptibility in adipocytes (50–63); (2) intersecting immune dysregulation including IFN suppression, hyper-inflammation and thrombosis, T-cell atrophy, and autoimmune disorders; and (3) metabolic disruption of glucose and lipid biosynthesis. In addition to the interaction of preexisting obesity with COVID-19, recent studies have divulged obesity**’**s effect on the recovery process and post-acute sequelae effect of COVID-19 in convalescent patients (**Figure 3**) (33, 37, 40, 48, 67, 68).

## Long COVID-19: Obesity and Immunometabolic Inception

Long COVID-19 or post-acute COVID-19 describes **“**persistent symptoms and/or delayed or long-term complications beyond 4 weeks from the onset of symptoms**”**, when the replication-competent SARS-CoV-2 is no longer isolated (155–158). Recent studies describe that long COVID sequelae often affect multiple organ systems, with diverse impact on function and quality of life post the acute disease (155–158). In one study of 4,182 COVID-19 cases, 588 (13.3%) reported symptoms lasting ≥28 days, 189 (4.5%) for ≥8 weeks, and 95 (2.3%) for ≥12 weeks. The common signs of long COVID included fatigue, headache, dyspnea, and anosmia and happened to be more likely with increased age, increased body mass index, and in females (158). Another study of 2,839 SARS-CoV-2-infected patients who survived the acute phase of COVID-19 without ICU admission (159) at 8 months post the acute COVID-19 disease found that the risk of hospital admission was 28% and 30% higher in patients with moderate and severe (BMI ≥ 35) obesity compared with patients with a normal body mass index (BMI < 30) (159). These findings indicate that moderate and severe obesity are associated with a greater risk of long COVID-19 (159). As no living virus has been detected during the long COVID-19, the persistent inflammation and other immunopathies from both obesity and remaining COVID-19 exasperation may provide an explanation. However, other viruses have been detected to be persistent in adipose tissues (127), which is hoped not to be the case for SARS-CoV-2 but warrants specific virological verification (53).

Finally, two recent studies concerning post-acute sequelae of COVID-19 reported a 6-month sequelae in two large cohorts of 30-day survivors of COVID-19 (160, 161). The first study analyzed the data of 236,379 UK patients diagnosed with COVID-19, concentrating on the neurological and psychiatric sequelae of COVID-19. They found substantial neurological and psychiatric morbidity (especially higher incidence of anxiety and psychotic disorders) 6 months after COVID-19 infection, showing that patients who had severe COVID-19 were at the greatest risks (160). Another study of 73,435 patients in the US using high dimensional analysis identified systemic incidence of 6-month sequelae, first in the respiratory system, and also in the nervous system and neurocognitive disorders, mental health disorders, metabolic disorders, cardiovascular disorders, gastrointestinal disorders, malaise, fatigue, musculoskeletal pain, and anemia (161). Previous studies have associated some neurological and psychotic disorders with early responses in obesity (7, 162), indicating that obesity and metabolic disorders could be one category of sequelae of long COVID-19 (159, 161). However, this does not necessarily imply that SARS-COV-2 has a direct obesogenic effect, rather that this is a consequence of the immunopathological interaction as proposed overall (6, 7).

## Inconclusive Remarks and Open Questions

Obesity and relevant metabolic comorbidities have been epidemic in recent decades, and are now compounded to threaten human health during the COVID-19 pandemic (1–18). As more and more studies have associated obesity and relevant disorders with a more severe prognosis of COVID-19, direct immunometabolic links underlying this association have emerged (10–18). Still, the major open questions include the following:

- Are obese individuals more susceptible to the viral infection or disease progress or both? Answers to this question will help to define the critical immune phase along viral infection stages in obese patients.
- Of the obesity-relevant BMI criterion or comorbidities, which serves as a better indicator for severe COVID-19 progress? Of note that the risk of obesity is often poorly defined due to a physical criterion based on BMI we currently use, and that looking closer at the differences in metabolic syndrome, obesity, and surrounding comorbidities could be a useful line of research.
- How important are adipocytes in SAR-CoV-2 infection and systemic spread in the body?
- Which are the key immunometabolic mediators and responsive cells underlying the association of obesity and severe COVID-19, and could they be used as biomarkers for the disease prognosis or cure in obese patients?
- What is the influence of obesity on the vaccine effect and COVID-19 progress, and how does it correlate to the immunometabolic dysregulation there?
- Do obesity and dysregulated immunity have an impact on the viral evolution and emergence of SARS-CoV-2 variants?
- Is obesity a sequela of the long COVID-19 (1–18)?

There are more questions than answers for most aspects underlying the interaction of obesity and COVID-19. Still, the most critical issue is to devise effective measures to protect obese COVID-19 patients (16–18). Given the significance of the coincidence of obesity in severe COVID-19 cases, we propose to explore the immunometabolic dysregulation at the interface of host metabolic and immune responses by directly using obese patient cohorts who experienced COVID-19 disease for studies in both acute and post-acute phases of the disease (10–18, 153–161). The therapeutic potential and awareness for drug invention to treat COVID-19 from a metabolic perspective was justified by a recent study at the immunometabolic interface. In this study, a disorder of lipid metabolism (phospholipidosis) was demonstrated to monotonically correlate with the antiviral efficacy of most repurposed drugs tested on COVID-19 (163). Therefore, early detection and proper intervention in lipid metabolism and relevant immunometabolic regulation should be examined critically for the invention and evaluation of candidate compounds or regimens to treat COVID-19 (162–164).

## Author Contributions

CK and MP contributed to reference collection, draft preparation, and proofreading. LM contributed to draft preparation and proofreading. YS supervises and conducts overall conceptualization, draft writing and figure drawing, review preparation, and funding acquisition. All authors contributed to the article and approved the submitted version.

## Funding

This work was supported by USDA NIFA Evans-Allen-1013186 and NIFA 2018-67016-28313 to YS, and in part through reagent sharing of NIFA AFRI 2020-67016-31347 and NSF-IOS-1831988 to YS.

## Conflict of Interest

The authors declare that the research was conducted in the absence of any commercial or financial relationships that could be construed as a potential conflict of interest.

## Publisher’s Note

All claims expressed in this article are solely those of the authors and do not necessarily represent those of their affiliated organizations, or those of the publisher, the editors and the reviewers. Any product that may be evaluated in this article, or claim that may be made by its manufacturer, is not guaranteed or endorsed by the publisher.

## References

[1] Obesity. World Health Organization (2021). Available at: tps://www.who.int/health-topics/obesity#tab=tab_1ht.

[2] Adult Obesity Facts. U.S: Center of Disease Control and Prevention (2021). Available at: https://www.cdc.gov/obesity/data/adult.html.

[3] Engin A. The Definition and Prevalence of Obesity and Metabolic Syndrome. Adv Exp Med Biol (2017) 960:1–17. doi: 10.1007/978-3-319-48382-5_1 28585193

[4] Andersen CJ Murphy KE Fernandez ML. Impact of Obesity and Metabolic Syndrome on Immunity. Adv Nutr (2016) 7(1):66–75. doi: 10.3945/an.115.010207 26773015 PMC4717890

[5] Tian Y Jennings J Gong Y Sang Y. Viral Infections and Interferons in the Development of Obesity. Biomolecules (2019) 9(11):726. doi: 10.3390/biom9110726 PMC6920831 31726661

[6] Sang Y Shields LE Sang ER Si H Pigg A Blecha F. Ileal Transcriptome Analysis in Obese Rats Induced by High-Fat Diets and an Adenoviral Infection. Int J Obes (2019) 43:2134–42. doi: 10.1038/s41366-019-0323-2 30670846

[7] Maier HE Lopez R Sanchez N Ng S Gresh L Ojeda S. Obesity Increases the Duration of Influenza A Virus Shedding in Adults. J Infect Dis (2018) 218(9):1378–82. doi: 10.1093/infdis/jiy370 PMC6151083 30085119

[8] Phung DT Wang Z Rutherford S Huang C Chu C. Body Mass Index and Risk of Pneumonia: A Systematic Review and Meta-Analysis. Obes Rev (2013) 14:839–57. doi: 10.1111/obr.12055 23800284

[9] Tan VPK Ngim CF Lee EZ Ramadas A Pong LY Ng JI. The Association Between Obesity and Dengue Virus (DENV) Infection in Hospitalised Patients. PloS One (2018) 13:e0200698. doi: 10.1371/journal.pone.0200698 30016369 PMC6049924

[10] Liu D Zhang T Wang Y Xia L. The Centrality of Obesity in the Course of Severe COVID-19. Front Endocrinol (Lausanne) (2021) 12:620566. doi: 10.3389/fendo.2021.620566 33776917 PMC7992974

[11] Report of the WHO-China Joint Mission on Coronavirus Disease 2019 (COVID-19). Available at: https://www.who.int/docs/default-source/coronaviruse/who-china-joint-mission-on-covid-19-final-report.pdf.

[12] Wadman M. Why Obesity Worsens COVID-19. Science (2020) 369(6509):1280–1. doi: 10.1126/science.369.6509.1280 32913079

[13] Senthilingam M. Covid-19 has Made the Obesity Epidemic Worse, But Failed to Ignite Enough Action. BMJ (2021) 372:n411. doi: 10.1136/bmj.n411 33664084

[14] Popkin BM Du S Green WD Beck MA Algaith T Herbst CH. Individuals With Obesity and COVID-19: A Global Perspective on the Epidemiology and Biological Relationships. Obes Rev (2020) 21(11):e13128. doi: 10.1111/obr.13128 32845580 PMC7461480

[15] Földi M Farkas N Kiss S Zádori N Váncsa S Szakó L. Obesity Is a Risk Factor for Developing Critical Condition in COVID-19 Patients: A Systematic Review and Meta-Analysis. Obes Rev (2020) 21(10):e13095. doi: 10.1111/obr.13095 32686331 PMC7404429

[16] Lavie CJ Coursin DB Long MT. The Obesity Paradox in Infections and Implications for COVID-19. Mayo Clin Proc (2021) 96(3):518–20. doi: 10.1016/j.mayocp.2021.01.014 PMC7835093 33673900

[17] Sharma A Garg A Rout A Lavie CJ. Association of Obesity With More Critical Illness in COVID-19. Mayo Clin Proc (2020) 95(9):2040–2. doi: 10.1016/j.mayocp.2020.06.046 PMC7330549 32861346

[18] Stefan N Birkenfeld AL Schulze MB Ludwig DS. Obesity and Impaired Metabolic Health in Patients With COVID-19. Nat Rev Endocrinol (2020) 16(7):341–2. doi: 10.1038/s41574-020-0364-6 PMC7187148 32327737

[19] Li X Zhong X Wang Y Zeng X Luo T Liu Q. Clinical Determinants of the Severity of COVID-19: A Systematic Review and Meta-Analysis. PloS One (2021) 16(5):e0250602. doi: 10.1371/journal.pone.0250602 33939733 PMC8092779

[20] Helvaci N Eyupoglu ND Karabulut E Yildiz BO. Prevalence of Obesity and Its Impact on Outcome in Patients With COVID-19: A Systematic Review and Meta-Analysis. Front Endocrinol (Lausanne) (2021) 12:598249. doi: 10.3389/fendo.2021.598249 33716962 PMC7947815

[21] Cottini M Lombardi C Berti A. Primary Care Physicians, ATS Province of Bergamo, Italy. Obesity Is a Major Risk Factor for Hospitalization in Community-Managed COVID-19 Pneumonia. Mayo Clin Proc (2021) 96(4):921–31. doi: 10.1016/j.mayocp.2021.01.021 PMC7859712 33814092

[22] Yu W Rohli KE Yang S Jia P. Impact of Obesity on COVID-19 Patients. J Diabetes Complications (2021) 35(3):107817. doi: 10.1016/j.jdiacomp.2020.107817 33358523 PMC7690270

[23] Fried MW Crawford JM Mospan AR Watkins SE Munoz Hernandez B Zink RC. Patient Characteristics and Outcomes of 11,721 Patients With COVID19 Hospitalized Across the United States. Clin Infect Dis (2021) 72:e558–65. doi: 10.1093/cid/ciaa1268. PMC7499515 32856034

[24] Bello-Chavolla OY Bahena-Lopez JP Antonio-Villa NE Vargas-Vazquez A Gonzalez-Diaz A Marquez-Salinas A. Predicting Mortality Due to SARS-CoV-2: A Mechanistic Score Relating Obesity and Diabetes to COVID-19 Outcomes in Mexico. J Clin Endocr Metab (2020) 105:2752–61. doi: 10.1210/clinem/dgaa346 PMC7313944 32474598

[25] Giannouchos TV Sussman RA Mier JM Poulas K Farsalinos K. Characteristics and Risk Factors for COVID-19 Diagnosis and Adverse Outcomes in Mexico: An Analysis of 89,756 Laboratory-Confirmed COVID-19 Cases. Eur Respir J (2020) 30:2002144. doi: 10.1183/13993003.02144-2020 PMC7397951 32732325

[26] Soares RCM Mattos LR Raposo LM. Risk Factors for Hospitalization and Mortality Due to COVID-19 in Espirito Santo State, Brazil. Am J Trop Med Hyg (2020) 103:1184–90. doi: 10.4269/ajtmh.20-0483 PMC7470570 32682453

[27] Hernandez-Galdamez DR Gonzalez-Block MA Romo-Duenas DK Lima-Morales R Hernandez-Vicente IA Lumbreras-Guzman M. Increased Risk of Hospitalization and Death in Patients With COVID-19 and Pre-Existing Noncommunicable Diseases and Modifiable Risk Factors in Mexico. Arch Med Res (2020) 51:683–9. doi: 10.1016/j.arcmed.2020.07.003 PMC7375298 32747155

[28] Yanover C Mizrahi B Kalkstein N Marcus K Akiva P Barer Y. What Factors Increase the Risk of Complications in SARS-CoV-2-Infected Patients? A Cohort Study in a Nationwide Israeli Health Organization. JMIR Public Health Surveill (2020) 6:e20872. doi: 10.2196/20872 32750009 PMC7451109

[29] Docherty AB Harrison EM Green CA Hardwick HE Pius R Norman L. Features of 20 133 UK Patients in Hospital With Covid-19 Using the ISARIC WHO Clinical Characterisation Protocol: Prospective Observational Cohort Study. BMJ (2020) 369:m1985. doi: 10.1136/bmj.m1985 32444460 PMC7243036

[30] Zhang X Lewis AM Moley JR Brestoff JR. A Systematic Review and Meta-Analysis of Obesity and COVID-19 Outcomes. Sci Rep (2021) 11:7193. doi: 10.1038/s41598-021-86694-1 33785830 PMC8009961

[31] Ho JSY Fernando DI Chan MY Sia CH. Obesity in COVID-19: A Systematic Review and Meta-Analysis. Ann Acad Med Singap (2020) 49(12):996–1008. doi: 10.47102/annals-acadmedsg.2020299 33463658

[32] Poly TN Islam MM Yang HC Lin MC Jian WS Hsu MH. Obesity and Mortality Among Patients Diagnosed With COVID-19: A Systematic Review and Meta-Analysis. Front Med (Lausanne) (2021) 8:620044. doi: 10.3389/fmed.2021.620044 33634150 PMC7901910

[33] Ramos-Casals M Brito-Zerón P Mariette X. Systemic and Organ-Specific Immune-Related Manifestations of COVID-19. Nat Rev Rheumatol (2021) 17(6):315–32. doi: 10.1038/s41584-021-00608-z PMC8072739 33903743

[34] Stefan N Birkenfeld AL Schulze MB. Global Pandemics Interconnected — Obesity, Impaired Metabolic Health and COVID-19. Nat Rev Endocrinol (2021) 17:135–49. doi: 10.1038/s41574-020-00462-1 33479538

[35] Cui X Zhao Z Zhang T Guo W Guo W Zheng J. A Systematic Review and Meta-Analysis of Children With Coronavirus Disease 2019 (COVID-19). J Med Virol (2021) 93(2):1057–69. doi: 10.1002/jmv.26398 PMC7436402 32761898

[36] Pousa PA Mendonça TSC Oliveira EA Simões-E-Silva AC. Extrapulmonary Manifestations of COVID-19 in Children: A Comprehensive Review and Pathophysiological Considerations. J Pediatr (Rio J) (2021) 97(2):116–39. doi: 10.1016/j.jped.2020.08.007 PMC7508521 32980319

[37] Ladhani SN Amin-Chowdhury Z Davies HG Aiano F Hayden I Lacy J. COVID-19 in Children: Analysis of the First Pandemic Peak in England. Arch Dis Child (2020) 105(12):1180–5. doi: 10.1136/archdischild-2020-320042 PMC7431771 32796006

[38] Christensen RAG Arneja J St. Cyr K Sturrock SL Brooks JD. The Association of Estimated Cardiorespiratory Fitness With COVID-19 Incidence and Mortality: A Cohort Study. PloS One (2021) 16(5):e0250508. doi: 10.1371/journal.pone.0250508 33951071 PMC8099071

[39] Gleeson LE Roche HM Sheedy FJ. Obesity, COVID-19 and Innate Immunometabolism. Br J Nutr (2021) 125(6):628–32. doi: 10.1017/S0007114520003529 PMC7520638 32892755

[40] Batabyal R Freishtat N Hill E Rehman M Freishtat R Koutroulis I. Metabolic Dysfunction and Immunometabolism in COVID-19 Pathophysiology and Therapeutics. Int J Obes (2021) 45:1163–9. doi: 10.1038/s41366-021-00804-7 PMC7961323 33727631

[41] Age, Sex, Existing Conditions of COVID-19 Cases and Deaths. (2021). Available at: https://www.worldometers.info/coronavirus/coronavirus-age-sex-demographics/.

[42] Courtney EP Goldenberg JL Boyd P. The Contagion of Mortality: A Terror Management Health Model for Pandemics. Br J Soc Psychol (2020) 59(3):607–17. doi: 10.1111/bjso.12392 PMC7323320 32557684

[43] Sanche S Lin YT Xu C Romero-Severson E Hengartner N Ke R. High Contagiousness and Rapid Spread of Severe Acute Respiratory Syndrome Coronavirus 2. Emerg Infect Dis (2020) 26(7):1470–7. doi: 10.3201/eid2607.200282 PMC7323562 32255761

[44] COVID-19 Pandemic Planning Scenarios. Available at: https://www.cdc.gov/coronavirus/2019-ncov/hcp/planning-scenarios.html.

[45] Scully EP Haverfield J Ursin RL Tannenbaum C Klein SL. Considering How Biological Sex Impacts Immune Responses and COVID-19 Outcomes. Nat Rev Immunol (2020) 20(7):442–7. doi: 10.1038/s41577-020-0348-8 PMC7288618 32528136

[46] Jutzeler CR Bourguignon L Weis CV Tong B Wong C Rieck B. Comorbidity, Clinical Signs and Symptoms, Laboratory Findings, Imaging Features, Treatment Strategies, and Outcomes in Adult and Pediatric Patients With COVID-19: A Systematic Review and Meta-Analysis. Travel Med Infect Dis (2020) 101825. doi: 10.1016/j.tmaid.2020.101825 32763496 PMC7402237

[47] Ehrenfeld M Tincani A Andreoli L Cattalini M Greenbaum A Kanduc D. Covid-19 and Autoimmunity. Autoimmun Rev (2020) 19(8):102597. doi: 10.1016/j.autrev.2020.102597 32535093 PMC7289100

[48] Lopez L Sang PC Tian Y Sang Y. Dysregulated Interferon Response Underlying Severe COVID-19. Viruses (2020) 12(12):1433. doi: 10.3390/v12121433 PMC7764122 33322160

[49] Rodríguez Y Novelli L Rojas M De Santis M Acosta-Ampudia Y Monsalve DM. Autoinflammatory and Autoimmune Conditions at the Crossroad of COVID-19. J Autoimmun (2020) 114:102506. doi: 10.1016/j.jaut.2020.102506 32563547 PMC7296326

[50] Murgolo N Therien AG Howell B Klein D Koeplinger K Lieberman LA. SARS-CoV-2 Tropism, Entry, Replication, and Propagation: Considerations for Drug Discovery and Development. PloS Pathog (2021) 17(2):e1009225. doi: 10.1371/journal.ppat.1009225 33596266 PMC7888651

[51] Sungnak W Huang N Bécavin C Berg M Queen R Litvinukova M. SARS-CoV-2 Entry Factors Are Highly Expressed in Nasal Epithelial Cells Together With Innate Immune Genes. Nat Med (2020) 26(5):681–7. doi: 10.1038/s41591-020-0868-6 PMC8637938 32327758

[52] Lamers MM Beumer J van der Vaart J Knoops K Puschhof J Breugem TI. SARS-CoV-2 Productively Infects Human Gut Enterocytes. Sci (2020) 369(6499):50–4. doi: 10.1126/science.abc1669 PMC7199907 32358202

[53] Farkash EA Wilson AM Jentzen JM. Ultrastructural Evidence for Direct Renal Infection With SARS-CoV-2. J Am Soc Nephrol (2020) 31(8):1683–7. doi: 10.1681/ASN.2020040432 PMC7460898 32371536

[54] Cardona Maya WD Du Plessis SS Velilla PA. SARS-CoV-2 and the Testis: Similarity With Other Viruses and Routes of Infection. Reprod BioMed Online (2020) 40(6):763–4. doi: 10.1016/j.rbmo.2020.04.009 PMC7162782 32362571

[55] Kusmartseva I Wu W Syed F Van Der Heide V Jorgensen M Joseph P. Expression of SARS-CoV-2 Entry Factors in the Pancreas of Normal Organ Donors and Individuals With COVID-19. Cell Metab (2020) 32(6):1041–51.e6. doi: 10.1016/j.cmet.2020.11.005 33207244 PMC7664515

[56] Coate KC Cha J Shrestha S Wang W Gonçalves LM Almaça J. SARS-CoV-2 Cell Entry Factors ACE2 and TMPRSS2 Are Expressed in the Microvasculature and Ducts of Human Pancreas But Are Not Enriched in β Cells. Cell Metab (2020) 32(6):1028–40.e4. doi: 10.1016/j.cmet.2020.11.006 33207245 PMC7664344

[57] Yao X-H He Z-C Li T-Y Zhang H-R Wang Y Mou H. Pathological Evidence for Residual SARS-CoV-2 in Pulmonary Tissues of a Ready-for-Discharge Patient. Cell Res (2020) 30:541–3. doi: 10.1038/s41422-020-0318-5 PMC7186763 32346074

[58] Gupte M Boustany-Kari CM Bharadwaj K Police S Thatcher S Gong MC. ACE2 Is Expressed in Mouse Adipocytes and Regulated by a High-Fat Diet. Am J Physiol Regul Integr Comp Physiol (2008) 295:R781–8. doi: 10.1152/ajpregu.00183.2008 PMC2536864 18650320

[59] de Melo GD Lazarini F Levallois S Hautefort C Michel V Larrous F. COVID-19-Related Anosmia Is Associated With Viral Persistence and Inflammation in Human Olfactory Epithelium and Brain Infection in Hamsters. Sci Transl Med (2021) 13(596):eabf8396. doi: 10.1126/scitranslmed.abf8396 33941622 PMC8158965

[60] Cantuti-Castelvetri L Ojha R Pedro LD Djannatian M Franz J Kuivanen S. Neuropilin-1 Facilitates SARS-CoV-2 Cell Entry and Infectivity. Sci (2020) 370(6518):856–60. doi: 10.1126/science.abd2985 PMC7857391 33082293

[61] Song E Zhang C Israelow B Lu-Culligan A Prado AV Skriabine S. Neuroinvasion of SARS-CoV-2 in Human and Mouse Brain. J Exp Med (2021) 218(3):e20202135. doi: 10.1084/jem.20202135 33433624 PMC7808299

[62] Wang Y Liu S Liu H Li W Lin F Jiang L. SARS-CoV-2 Infection of the Liver Directly Contributes to Hepatic Impairment in Patients With COVID-19. J Hepatol (2020) 73(4):807–16. doi: 10.1016/j.jhep.2020.05.002 PMC7211738 32437830

[63] Synowiec A Szczepański A Barreto-Duran E Lie LK Pyrc K. Severe Acute Respiratory Syndrome Coronavirus 2 (SARS-CoV-2): A Systemic Infection. Clin Microbiol Rev (2021) 34(2):e00133–20. doi: 10.1128/CMR.00133-20 PMC7849242 33441314

[64] Alifano M Alifano P Forgez P Iannelli A. Renin-Angiotensin System at the Heart of COVID-19 Pandemic. Biochimie (2020) 174:30–3. doi: 10.1016/j.biochi.2020.04.008 PMC7161528 32305506

[65] Naik GOA. COVID-19 and the Renin-Angiotensin-Aldosterone System. Clin Infect Dis (2021) 72(6):1105–7. doi: 10.1093/cid/ciaa818 PMC7337648 32561913

[66] Rysz S Al-Saadi J Sjöström A Farm M Campoccia Jalde F Plattén M. COVID-19 Pathophysiology May Be Driven by an Imbalance in the Renin-Angiotensin-Aldosterone System. Nat Commun (2021) 12:2417. doi: 10.1038/s41467-021-22713-z 33893295 PMC8065208

[67] Schultze JL Aschenbrenner AC. COVID-19 and the Human Innate Immune System. Cell (2021) 184(7):1671–92. doi: 10.1016/j.cell.2021.02.029 PMC7885626 33743212

[68] Paces J Strizova Z Smrz D Cerny J. COVID-19 and the Immune System. Physiol Res (2020) 69(3):379–88. doi: 10.33549/physiolres.934492 PMC8648321 32469225

[69] Karki R Sharma BR Tuladhar S Williams EP Zalduondo L Samir P. Synergism of TNF-α and IFN-γ Triggers Inflammatory Cell Death, Tissue Damage, and Mortality in SARS-CoV-2 Infection and Cytokine Shock Syndromes. Cell (2021) 184(1):149–68.e17. doi: 10.1016/j.cell.2020.11.025 33278357 PMC7674074

[70] Redinger RN. The Pathophysiology of Obesity and Its Clinical Manifestations. Gastroenterol Hepatol (NY) (2007) 3(11):856–63. PMC3104148 21960798

[71] Zmora N Bashiardes S Levy M Elinav E. The Role of the Immune System in Metabolic Health and Disease. Cell Metab (2017) 25(3):506–21. doi: 10.1016/j.cmet.2017.02.006 28273474

[72] Francisco V Pino J Campos-Cabaleiro V Ruiz-Fernández C Mera A Gonzalez-Gay MA. Obesity, Fat Mass and Immune System: Role for Leptin. Front Physiol (2018) 9:640. doi: 10.3389/fphys.2018.00640 29910742 PMC5992476

[73] Morange PE Alessi MC. Thrombosis in Central Obesity and Metabolic Syndrome: Mechanisms and Epidemiology. Thromb Haemost (2013) 110(4):669–80. doi: 10.1160/TH13-01-0075 23765199

[74] Vilahur G Ben-Aicha S Badimon L. New Insights Into the Role of Adipose Tissue in Thrombosis. Cardiovasc Res (2017) 113(9):1046–54. doi: 10.1093/cvr/cvx086 28472252

[75] Medina G Vera-Lastra O Peralta-Amaro AL Jiménez-Arellano MP Saavedra MA Cruz-Domínguez MP. Metabolic Syndrome, Autoimmunity and Rheumatic Diseases. Pharmacol Res (2018) 133:277–88. doi: 10.1016/j.phrs.2018.01.009 29382608

[76] Mok CC. Metabolic Syndrome and Systemic Lupus Erythematosus: The Connection. Expert Rev Clin Immunol (2019) 15(7):765–75. doi: 10.1080/1744666X.2019.1620601 31094570

[77] Liu TC Kern JT Jain U Sonnek NM Xiong S Simpson KF. Western Diet Induces Paneth Cell Defects Through Microbiome Alterations and Farnesoid X Receptor and Type I Interferon Activation. Cell Host Microbe (2021) 29(6):988–1001.e6. doi: 10.1016/j.chom.2021.04.004 34010595 PMC8192497

[78] Strissel KJ Stancheva Z Miyoshi H Perfield JW 2nd DeFuria J Jick Z. Adipocyte Death, Adipose Tissue Remodeling, and Obesity Complications. Diabetes (2007) 56(12):2910–8. doi: 10.2337/db07-0767 17848624

[79] Kuroda M Sakaue H. Adipocyte Death and Chronic Inflammation in Obesity. J Med Invest (2017) 64(3.4):193–6. doi: 10.2152/jmi.64.193 28954980

[80] Liu Z Wu KKL Jiang X Xu A Cheng KKY. The Role of Adipose Tissue Senescence in Obesity- and Ageing-Related Metabolic Disorders. Clin Sci (Lond) (2020) 134(2):315–30. doi: 10.1042/CS20190966 31998947

[81] Ogrodnik M Zhu Y Langhi LGP Tchkonia T Krüger P Fielder E. Obesity-Induced Cellular Senescence Drives Anxiety and Impairs Neurogenesis. Cell Metab (2019) 29(5):1061–77.e8. doi: 10.1016/j.cmet.2018.12.008 30612898 PMC6509403

[82] Shirakawa K Yan X Shinmura K Endo J Kataoka M Katsumata Y. Obesity Accelerates T Cell Senescence in Murine Visceral Adipose Tissue. J Clin Invest (2016) 126(12):4626–39. doi: 10.1172/JCI88606 PMC5127667 27820698

[83] Smith U Li Q Rydén M Spalding KL. Cellular Senescence and Its Role in White Adipose Tissue. Int J Obes (2021) 45:934–43. doi: 10.1038/s41366-021-00757-x 33510393

[84] Camell CD Yousefzadeh MJ Zhu Y Langhi Prata LGP Huggins MA Pierson M. Senolytics Reduce Coronavirus-Related Mortality in Old Mice. Science (2021) 373:eabe4832. doi: 10.1126/science.abe4832 34103349 PMC8607935

[85] Fasshauer M Blüher M. Adipokines in Health and Disease. Trends Pharmacol Sci (2015) 36(7):461–70. doi: 10.1016/j.tips.2015.04.014 26022934

[86] Lelis DF Freitas DF Machado AS Crespo TS Santos SHS. Angiotensin-(1-7), Adipokines and Inflammation. Metab (2019) 95:36–45. doi: 10.1016/j.metabol.2019.03.006 30905634

[87] Weiss R Dziura J Burgert TS Tamborlane WV Taksali SE Yeckel CW. Obesity and the Metabolic Syndrome in Children and Adolescents. N Engl J Med (2004) 350(23):2362–74. doi: 10.1056/NEJMoa031049 15175438

[88] Tilg H Moschen AR. Adipocytokines: Mediators Linking Adipose Tissue, Inflammation and Immunity. Nat Rev Immunol (2006) 6(10):772–83. doi: 10.1038/nri1937 16998510

[89] Lago F Dieguez C Gómez-Reino J Gualillo O. Adipokines as Emerging Mediators of Immune Response and Inflammation. Nat Rev Rheumatol (2007) 3(12):716–24. doi: 10.1038/ncprheum0674 18037931

[90] Ouchi N Parker JL Lugus JJ Walsh K. Adipokines in Inflammation and Metabolic Disease. Nat Rev Immunol (2011) 11(2):85–97. doi: 10.1038/nri2921 21252989 PMC3518031

[91] La Cava A. Leptin in Inflammation and Autoimmunity. Cytokine (2017) 98:51–8. doi: 10.1016/j.cyto.2016.10.011 PMC5453851 27916613

[92] Abella V Scotece M Conde J Pino J Gonzalez-Gay MA Gómez-Reino JJ. Leptin in the Interplay of Inflammation, Metabolism and Immune System Disorders. Nat Rev Rheumatol (2017) 13(2):100–9. doi: 10.1038/nrrheum.2016.209 28053336

[93] van Deursen JM. The Role of Senescent Cells in Ageing. Nature (2014) 509(7501):439–46. doi: 10.1038/nature13193 PMC4214092 24848057

[94] Kanneganti TD Dixit VD. Immunological Complications of Obesity. Nat Immunol (2012) 13(8):707–12. doi: 10.1038/ni.2343 22814340

[95] Yang H Youm YH Vandanmagsar B Rood J Kumar KG Butler AA. Obesity Accelerates Thymic Aging. Blood (2009) 114:3803–12. doi: 10.1182/blood-2009-03-213595 PMC2773495 19721009

[96] Ghanim H Aljada A Hofmeyer D Syed T Mohanty P Dandona P. Circulating Mononuclear Cells in the Obese Are in a Proinflammatory State. Circulation (2004) 110:1564–71. doi: 10.1161/01.CIR.0000142055.53122.FA 15364812

[97] Karlsson EA Sheridan PA Beck MA. Diet-Induced Obesity in Mice Reduces the Maintenance of Influenza-Specific CD8+ Memory T Cells. J Nutr (2010) 140:1691–7. doi: 10.3945/jn.110.123653 PMC2924599 20592105

[98] White PJ Arita M Taguchi R Kang JX Marette A. Transgenic Restoration of Long-Chain N-3 Fatty Acids in Insulin Target Tissues Improves Resolution Capacity and Alleviates Obesity-Linked Inflammation and Insulin Resistance in High-Fat-Fed Mice. Diabetes (2010) 59:3066–73. doi: 10.2337/db10-0054 PMC2992767 20841610

[99] Jiang Y Wei X Guan J Qin S Wang Z Lu H. COVID-19 Pneumonia: CD8+ T and NK Cells Are Decreased in Number But Compensatory Increased in Cytotoxic Potential. Clin Immunol (2020) 218:108516. doi: 10.1016/j.clim.2020.108516 32574709 PMC7305921

[100] Jouan Y Guillon A Gonzalez L Perez Y Boisseau C Ehrmann S. Phenotypical and Functional Alteration of Unconventional T Cells in Severe COVID-19 Patients. J Exp Med (2020) 217(12):e20200872. doi: 10.1084/jem.20200872 32886755 PMC7472174

[101] Flament H Rouland M Beaudoin L Toubal A Bertrand L Lebourgeois S. Outcome of SARS-CoV-2 Infection Is Linked to MAIT Cell Activation and Cytotoxicity. Nat Immunol (2021) 22(3):322–35. doi: 10.1038/s41590-021-00870-z 33531712

[102] Parrot T Gorin JB Ponzetta A Maleki KT Kammann T Emgård J. MAIT Cell Activation and Dynamics Associated With COVID-19 Disease Severity. Sci Immunol (2020) 5(51):eabe1670. doi: 10.1126/sciimmunol.abe1670 32989174 PMC7857393

[103] Maucourant C Filipovic I Ponzetta A Aleman S Cornillet M Hertwig L. Natural Killer Cell Immunotypes Related to COVID-19 Disease Severity. Sci Immunol (2020) 5(50):eabd6832. doi: 10.1126/sciimmunol.abd6832 32826343 PMC7665314

[104] Wilk AJ Rustagi A Zhao NQ Roque J Martínez-Colón GJ McKechnie JL. A Single-Cell Atlas of the Peripheral Immune Response in Patients With Severe COVID-19. Nat Med (2020) 26(7):1070–6. doi: 10.1038/s41591-020-0944-y PMC7382903 32514174

[105] Silvin A Chapuis N Dunsmore G Goubet AG Dubuisson A Derosa L. Elevated Calprotectin and Abnormal Myeloid Cell Subsets Discriminate Severe From Mild COVID-19. Cell (2020) 182(6):1401–1418.e18. 32810439 10.1016/j.cell.2020.08.002 PMC7405878

[106] Schulte-Schrepping J Reusch N Paclik D Baßler K Schlickeiser S Zhang B. Severe COVID-19 Is Marked by a Dysregulated Myeloid Cell Compartment. Cell (2020) 182(6):1419–40.e23. doi: 10.1016/j.cell.2020.08.001 32810438 PMC7405822

[107] Rydyznski Moderbacher C Ramirez SI Dan JM Grifoni A Hastie KM Weiskopf D. Antigen-Specific Adaptive Immunity to SARS-CoV-2 in Acute COVID-19 and Associations With Age and Disease Severity. Cell (2020) 183(4):996–1012.e19. doi: 10.1016/j.cell.2020.09.038 33010815 PMC7494270

[108] Bergamaschi L Mescia F Turner L Hanson AL Kotagiri P Dunmore BJ. Longitudinal Analysis Reveals That Delayed Bystander CD8+ T Cell Activation and Early Immune Pathology Distinguish Severe COVID-19 From Mild Disease. Immunity (2021) 54:1257–75.e8. doi: 10.1016/j.immuni.2021.05.010. PMC8125900 34051148

[109] Dugan HL Stamper CT Li L Changrob S Asby NW Halfmann PJ. Profiling B Cell Immunodominance After SARS-CoV-2 Infection Reveals Antibody Evolution to Non-Neutralizing Viral Targets. Immunity (2021) 54:1290–303.e7. doi: 10.1016/j.immuni.2021.05.001. PMC8101792 34022127

[110] Murugan AT Sharma G. Obesity and Respiratory Diseases. Chron Resp Dis (2008) 5(4):233–42. doi: 10.1177/1479972308096978 19029235

[111] Elliot JG Donovan GM Wang K Green F James AL Noble PB. Fatty Airways: Implications for Obstructive Disease. Eur Respir J (2019) 54(6):1900857. doi: 10.1183/13993003.00857-2019 31624112

[112] Leone N Courbon D Thomas F Bean K Jego B Leynaert B. Lung Function Impairment and Metabolic Syndrome: The Critical Role of Abdominal Obesity. Am J Respir Crit Care Med (2009) 179(6):509–16. doi: 10.1164/rccm.200807-1195OC 19136371

[113] Sideleva O Suratt BT Black KE Tharp WG Pratley RE Forgione P. Obesity and Asthma: An Inflammatory Disease of Adipose Tissue Not the Airway. Am J Respir Crit Care Med (2012) 186(7):598–605. doi: 10.1164/rccm.201203-0573OC 22837379 PMC3480522

[114] Watanabe K Suzukawa M Arakawa S Kobayashi K Igarashi S Tashimo H. Leptin Enhances Cytokine/Chemokine Production by Normal Lung Fibroblasts by Binding to Leptin Receptor. Allergol Int (2019) 68S:S3–8. doi: 10.1016/j.alit.2019.04.002 31029506

[115] Bourgonje AR Abdulle AE Timens W Hillebrands JL Navis GJ Gordijn SJ. Angiotensin-Converting Enzyme 2 (ACE2), SARS-CoV-2 and the Pathophysiology of Coronavirus Disease 2019 (COVID-19). J Pathol (2020) 251(3):228–48. doi: 10.1002/path.5471 PMC7276767 32418199

[116] Dhont S Derom E Van Braeckel E Depuydt P Lambrecht BN. The Pathophysiology of 'Happy' Hypoxemia in COVID-19. Respir Res (2020) 21(1):198. doi: 10.1186/s12931-020-01462-5 32723327 PMC7385717

[117] Serebrovska ZO Chong EY Serebrovska TV Tumanovska LV Xi L. Hypoxia, HIF-1α, and COVID-19: From Pathogenic Factors to Potential Therapeutic Targets. Acta Pharmacol Sin (2020) 41(12):1539–46. doi: 10.1038/s41401-020-00554-8 PMC7588589 33110240

[118] Foldi M Farkas N Kiss S Dembrovszky F Szakacs Z Balasko M. Visceral Adiposity Elevates the Risk of Critical Condition in COVID-19: A Systematic Review and Meta-Analysis. Obes (Silver Spring) (2020) 29:521–8. doi: 10.1002/oby.23096 PMC7753720 33263191

[119] Pereira-Santos M Costa PR Assis AM Santos CA Santos DB. Obesity and Vitamin D Deficiency: A Systematic Review and Meta-Analysis. Obes Rev (2015) 16(4):341–9. doi: 10.1111/obr.12239 25688659

[120] Mohan M Cherian JJ Sharma A. Exploring Links Between Vitamin D Deficiency and COVID-19. PloS Pathog (2020) 16(9):e1008874. doi: 10.1371/journal.ppat.1008874 32946517 PMC7500624

[121] Zang R Case JB Yutuc E Ma X Shen S Gomez Castro MF. Cholesterol 25-Hydroxylase Suppresses SARS-CoV-2 Replication by Blocking Membrane Fusion. Proc Natl Acad Sci USA (2020) 117(50):32105–13. doi: 10.1073/pnas.2012197117 PMC7749331 33239446

[122] Maddaloni E Cavallari I Napoli N Conte C. Vitamin D and Diabetes Mellitus. Front Horm Res (2018) 50:161–76. doi: 10.1159/000486083 29597238

[123] Bonaventura A Vecchié A Dagna L Martinod K Dixon DL Van Tassell BW. Endothelial Dysfunction and Immunothrombosis as Key Pathogenic Mechanisms in COVID-19. Nat Rev Immunol (2021) 21(5):319–29. doi: 10.1038/s41577-021-00536-9 PMC8023349 33824483

[124] Becker RC. COVID-19-Associated Vasculitis and Vasculopathy. J Thromb Thrombolysis (2020) 50(3):499–511. doi: 10.1007/s11239-020-02230-4 32700024 PMC7373848

[125] Pasquarelli-do-Nascimento G Braz-de-Melo HA Faria SS Santos IO Kobinger GP Magalhães KG. Hypercoagulopathy and Adipose Tissue Exacerbated Inflammation May Explain Higher Mortality in COVID-19 Patients With Obesity. Front Endocrinol (Lausanne) (2020) 11:530. doi: 10.3389/fendo.2020.00530 32849309 PMC7399077

[126] Bastard P Rosen LB Zhang Q Michailidis E Hoffmann HH Zhang Y. Auto-Antibodies Against Type I IFNs in Patients With Life-Threatening COVID-19. Science (2020) 370:eabd4585. doi: 10.1126/science.abd4585 32972996 PMC7857397

[127] Ryan PM Caplice NM. Is Adipose Tissue a Reservoir for Viral Spread, Immune Activation, and Cytokine Amplification in Coronavirus Disease 2019? . Obes (Silver Spring) (2020) 28(7):1191–4. doi: 10.1002/oby.22843 PMC7264526 32314868

[128] Perico L Benigni A Casiraghi F Ng LFP Renia L Remuzzi G. Immunity, Endothelial Injury and Complement-Induced Coagulopathy in COVID-19. Nat Rev Nephrol (2021) 17(1):46–64. doi: 10.1038/s41581-020-00357-4 33077917 PMC7570423

[129] Sang ER Tian Y Miller LC Sang Y. Epigenetic Evolution of ACE2 and IL-6 Genes: Non-Canonical Interferon-Stimulated Genes Correlate to COVID-19 Susceptibility in Vertebrates. Genes (Basel) (2021) 12(2):154. doi: 10.3390/genes12020154 33503821 PMC7912275

[130] Chlamydas S Papavassiliou AG Piperi C. Epigenetic Mechanisms Regulating COVID-19 Infection. Epigenetics (2020) 30:1–8. doi: 10.1080/15592294.2020.1796896 PMC7901548 32686577

[131] Sawalha AH Zhao M Coit P Lu Q. Epigenetic Dysregulation of ACE2 and Interferon-Regulated Genes Might Suggest Increased COVID-19 Susceptibility and Severity in Lupus Patients. Clin Immunol (2020) 215:108410. doi: 10.1016/j.clim.2020.108410 32276140 PMC7139239

[132] Barrat FJ Crow MK Ivashkiv LB. Interferon Target-Gene Expression and Epigenomic Signatures in Health and Disease. Nat Immunol (2019) 20(12):1574–83. doi: 10.1038/s41590-019-0466-2 PMC7024546 31745335

[133] Sette A Crotty S. Adaptive Immunity to SARS-CoV-2 and COVID-19. Cell (2021) 184(4):861–80. doi: 10.1016/j.cell.2021.01.007 PMC7803150 33497610

[134] Mesev EV LeDesma RA Ploss A. Decoding Type I and III Interferon Signalling During Viral Infection. Nat Microbiol (2019) 4:914–24. doi: 10.1038/s41564-019-0421-x PMC6554024 30936491

[135] Hadjadj J Yatim N Barnabei L Corneau A Boussier J Smith N. Impaired Type I Interferon Activity and Inflammatory Responses in Severe COVID-19 Patients. Sci (2020) 369(6504):718–24. doi: 10.1126/science.abc6027 PMC7402632 32661059

[136] Blanco-Melo D Nilsson-Payant BE Liu WC Uhl S Hoagland D Møller R. Imbalanced Host Response to SARS-CoV-2 Drives Development of COVID-19. Cell (2020) 181(5):1036–45.e9. doi: 10.1016/j.cell.2020.04.026 32416070 PMC7227586

[137] Lee JS Park S Jeong HW Ahn JY Choi SJ Lee H. Immunophenotyping of COVID-19 and Influenza Highlights the Role of Type I Interferons in Development of Severe COVID-19. Sci Immunol (2020) 5:eabd1554. doi: 10.1126/sciimmunol.abd1554 32651212 PMC7402635

[138] Lucas C Wong P Klein J Castro TBR Silva J Sundaram M. Longitudinal Analyses Reveal Immunological Misfiring in Severe COVID-19. Nature (2020) 584(7821):463–9. doi: 10.1038/s41586-020-2588-y PMC7477538 32717743

[139] Sa Ribero M Jouvenet N Dreux M Nisole S. Interplay Between SARS-CoV-2 and the Type I Interferon Response. PloS Pathog (2020) 16(7):e1008737. doi: 10.1371/journal.ppat.1008737 32726355 PMC7390284

[140] Pairo-Castineira E Clohisey S Klaric L Bretherick AD Rawlik K Pasko D. Genetic Mechanisms of Critical Illness in COVID-19. Nat (2021) 591(7848):92–8. doi: 10.1038/s41586-020-03065-y 33307546

[141] McCoy K Peterson A Tian Y Sang Y. Immunogenetic Association Underlying Severe COVID-19. Vaccines (Basel) (2020) 8(4):700. doi: 10.3390/vaccines8040700 PMC7711778 33233531

[142] Zhang Y Chen Y LI Y Huang F Luo B Yuan Y. The ORF8 Protein of SARS-CoV-2 Mediates Immune Evasion Through Down-Regulating MHC-Ι. Proc Natl Acad Sci USA (2021) 118:e2024202118. doi: 10.1073/pnas.2024202118. 34021074 PMC8201919

[143] Taefehshokr N Taefehshokr S Hemmat N Heit B. Covid-19: Perspectives on Innate Immune Evasion. Front Immunol (2020) 11:580641. doi: 10.3389/fimmu.2020.580641 33101306 PMC7554241

[144] McCormick PJ tina JA Bonifacino JS. Involvement of Clathrin and AP-2 in the Trafficking of MHC Class II Molecules to Antigen-Processing Compartments. Proc Natl Acad Sci USA (2005) 102:7910. doi: 10.1073/pnas.0502206102 15911768 PMC1138261

[145] Gordon DE Jang GM Bouhaddou M Xu J Obernier K White KM. A SARS-CoV-2 Protein Interaction Map Reveals Targets for Drug Repurposing. Nat (2020) 583(7816):459–68. doi: 10.1038/s41586-020-2286-9 PMC7431030 32353859

[146] Braun J Loyal L Frentsch M Wendisch D Georg P Kurth F. SARS-CoV-2-Reactive T Cells in Healthy Donors and Patients With COVID-19. Nature (2020) 587:270–4. doi: 10.1038/s41586-020-2598-9 32726801

[147] Tan AT Linster M Tan CW Le Bert N Chia WN Kunasegaran K. Early Induction of Functional SARS-CoV-2-Specific T Cells Associates With Rapid Viral Clearance and Mild Disease in COVID-19 Patients. Cell Rep (2021) 34(6):108728. doi: 10.1016/j.celrep.2021.108728 33516277 PMC7826084

[148] Zhang Q Bastard P Liu Z Le Pen J Moncada-Velez M Chen J. Inborn Errors of Type I IFN Immunity in Patients With Life-Threatening COVID-19. Science (2020) 370:eabd4570. doi: 10.1126/science.abd4570. 32972995 PMC7857407

[149] Favre GA Esnault VL Van Obberghen E. Modulation of Glucose Metabolism by the Renin-Angiotensin-Aldosterone System. Am J Physiol Endocrinol Metab (2015) 308(6):E435–49. doi: 10.1152/ajpendo.00391.2014 25564475

[150] Song JW Lam SM Fan X Cao WJ Wang SY Tian H. Omics-Driven Systems Interrogation of Metabolic Dysregulation in COVID-19 Pathogenesis. Cell Metab (2020) 32(2):188–202.e5. doi: 10.1016/j.cmet.2020.06.016 32610096 PMC7311890

[151] Maceyka M Spiegel S. Sphingolipid Metabolites in Inflammatory Disease. Nat (2014) 510(7503):58–67. doi: 10.1038/nature13475 PMC4320971 24899305

[152] Hannun YA Obeid LM. Sphingolipids and Their Metabolism in Physiology and Disease. Nat Rev Mol Cell Biol (2018) 19(3):175–91. doi: 10.1038/nrm.2017.107 PMC5902181 29165427

[153] Yu B Li C Sun Y Wang DW. Insulin Treatment Is Associated With Increased Mortality in Patients With COVID-19 and Type 2 Diabetes. Cell Metab (2021) 33(1):65–77.e2. doi: 10.1016/j.cmet.2020.11.014 33248471 PMC7682421

[154] Hoffmann HH Sánchez-Rivera FJ Schneider WM Luna JM Soto-Feliciano YM Ashbrook AW. Functional Interrogation of a SARS-CoV-2 Host Protein Interactome Identifies Unique and Shared Coronavirus Host Factors. Cell Host Microbe (2021) 29(2):267–80.e5. doi: 10.1016/j.chom.2020.12.009 33357464 PMC7833927

[155] Marshall M. The Four Most Urgent Questions About Long COVID. Nat (2021) 594(7862):168–70. doi: 10.1038/d41586-021-01511-z 34108700

[156] Davis HE Assaf GS McCorkell L Wei H Low RJ Re’em Y. Characterizing Long COVID in an International Cohort: 7 Months of Symptoms and Their Impact. EClinicalMedicine (2021) 38:101019. doi: 10.1016/j.eclinm.2021.101019 34308300 PMC8280690

[157] Nalbandian A Sehgal K Gupta A Madhavan MV McGroder C Stevens JS. Post-Acute COVID-19 Syndrome. Nat Med (2021) 27:601–15. doi: 10.1038/s41591-021-01283-z PMC8893149 33753937

[158] Sudre CH Murray B Varsavsky T Graham MS Penfold RS Bowyer RC. Attributes and Predictors of Long COVID. Nat Med (2021) 27:626–31. doi: 10.1038/s41591-021-01292-y PMC7611399 33692530

[159] Aminian A Bena J Pantalone KM Burguera B. Association of Obesity With Postacute Sequelae of COVID-19. Diabetes Obes Metab (2021) 23(10):2183–8. doi: 10.1111/dom.14454 PMC8239834 34060194

[160] Al-Aly Z Xie Y Bowe B. High-Dimensional Characterization of Post-Acute Sequelae of COVID-19. Nat (2021) 594(7862):259–64. doi: 10.1038/s41586-021-03553-9 33887749

[161] Taquet M Geddes JR Husain M Luciano S Harrison PJ. 6-Month Neurological and Psychiatric Outcomes in 236 379 Survivors of COVID-19: A Retrospective Cohort Study Using Electronic Health Records. Lancet Psychiatry (2021) 8(5):416–27. doi: 10.1016/S2215-0366(21)00084-5 PMC8023694 33836148

[162] Wang S Li W Hui H Tiwari SK Zhang Q Croker BA. Cholesterol 25-Hydroxylase Inhibits SARS-CoV-2 and Other Coronaviruses by Depleting Membrane Cholesterol. EMBO J (2020) 39(21):e106057. doi: 10.15252/embj.2020106057 32944968 PMC7537045

[163] Tummino TA Rezelj VV Fischer B Fischer A O'Meara MJ Monel B. Drug-Induced Phospholipidosis Confounds Drug Repurposing for SARS-CoV-2. Science (2021) 373:541–7. doi: 10.1126/science.abi4708 PMC8501941 34326236

[164] Yuan S Chan CC Chik KK Tsang JO Liang R Cao J. Broad-Spectrum Host-Based Antivirals Targeting the Interferon and Lipogenesis Pathways as Potential Treatment Options for the Pandemic Coronavirus Disease 2019 (COVID-19). Viruses (2020) 12(6):628. doi: 10.3390/v12060628 PMC7354423 32532085

